# Supplementary material for: A new Meckel’s cartilage from the Devonian Hangenberg black shale in Morocco and its position in chondrichthyan jaw morphospace
Source: PeerJ. 2022 Dec 21;10:e14418. doi: 10.7717/peerj.14418 (PMC9789696; doi:10.7717/peerj.14418)
Supplement: Supplemental Information 1 [file peerj-10-14418-s001.docx]

**Appendix**

Table 1: List of all taxa/ specimens used in the Fourier Analysis and their references

| Species | Reference |
| --- | --- |
| 1. New HBS specimen | **PIMUZ A/I 5139**; Stored at the Paleontological Institute and Museum in Zürich, Switzerland |
| 2. *Ischnacanthus* sp. | **Dearden RP, Giles S. 2021**. Diverse stem-chondrichthyan oral structures and evidence for an independently acquired acanthodid dentition. *Royal Society Open Science* **8:**210822 DOI 10.1098/rsos.210822. |
| 3. *Climatius reticulatus* | **Burrow CJ, Davidson RG, Den Blaauwen JL, Newman MJ. 2015.** Revision of *Climatius reticulatus* Agassiz, 1844 (Acanthodii, Climatiidae), from the Lower Devonian of Scotland, based on new histological and morphological data. *Journal of Vertebrate*  *Paleontology* **35(3):**e913421 DOI 10.1080/02724634.2014.913421. |
| 4. *Latviacanthus ventspilsensis* | **Schultze HP, Zidek NJ. 1982**. Ein primitiver Acanthodier (pisces) aus dem Unterdevon Lettlands. *Paläontologische Zeitschrift* **56(1-2):**95_105 DOI 10.1007/BF02988788. |
| 5. *Ptomacanthus anglicus* | **Brazeau MD. 2012**. A revision of the anatomy of the Early Devonian jawed vertebrate *Ptomacanthus anglicus* Miles. *Palaeontology* **55(2):**355_367 DOI 10.1111/j.1475-4983.2012.01130.x. |
| 6. *Acanthodopsis* sp. | **Dearden RP, Giles S. 2021**. Diverse stem-chondrichthyan oral structures and evidence for an independently acquired acanthodid dentition. *Royal Society Open Science* **8**:210822 DOI 10.1098/rsos.210822. |
| 7. *Tetanopsyrus lindoei* | **Hanke GF, Davis SP, Wilson MVH. 2001**. New Species of the Acanthodian Genus *Tetanopsyrus* from Northern Canada, and Comments on Related Taxa. *Journal of Vertebrate Paleontology* **21(4):**740_753 DOI 10.1671/0272 4634(2001)021[0740:NSOTAG]2.0.CO;2. |
| 8. *Cheiracanthus grandispinus* | **Burrow CJ, Blaauwen JD, Newman M. 2020**. A redescription of the three longest-known species of the acanthodian *Cheiracanthus* from the Middle Devonian of Scotland. *Palaeontologia Electronica* **23(1):**a15. |
| 9. *Cheiracanthus murchisoni* | **Burrow CJ, Blaauwen JD, Newman M. 2020.** A redescription of the three longest-known species of the acanthodian *Cheiracanthus* from the Middle Devonian of Scotland. *Palaeontologia Electronica* **23(1**):a15. |
| 10. *Halimacanthodes ahlbergi* | **Burrow CJ, Trinajstic K, Long J. 2012**. First acanthodian from the Upper Devonian (Frasnian) Gogo Formation, Western Australia. *Historical Biology* **24(4):**349_357 DOI 10.1080/08912963.2012.660150. |
| 11. *Acanthodes bronni* | **Davis SP, Finarelli JA, Coates MI. 2012.** *Acanthodes* and shark-like conditions in the last common ancestor of modern gnathostomes. Nature **486(7402):**247_250 DOI 10.1038/nature11080. |
| 12. *Pucapampella* sp. | **Maisey, J. G., Janvier, P., Pradel, A., Denton, J. S. S., Bronson, A., Miller, R., & Burrow, C. J. (2019).** *Doliodus* and pucapampellids: contrasting perspectives on stem chondrichthyan morphology. Evolution and development of fishes, 87-109. DOI:[10.1017/9781316832172.006](http://dx.doi.org/10.1017/9781316832172.006) |
| 13. *Gladbachus adentatus* | **Coates MI, Finarelli JA, Sansom IJ, Andreev PS, Criswell KE, Tietjen K, Rivers ML, La Riviere PJ. 2018**. An early chondrichthyan and the evolutionary assembly of a shark body plan. *Proceedings. Biological sciences* **285**:20172418. DOI 10.1098/rspb.2017.2418. |
| 14. *Kathemacanthus rosulentus* | **Hanke GF, Wilson MVH. 2006**. Anatomy of the early Devonian acanthodian *Brochoadmones milesi* based on nearly complete body fossils, with comments on the evolutionand development of paired fins. *Journal of Vertebrate Paleontology* **26(3):**526_537  DOI 10.1671/0272-4634(2006)26[526:AOTEDA]2.0.CO;2. |
| 15. *Gogoselachus lynbeazleyae* | **Long JA, Burrow CJ, Ginter M, Maisey JG, Trinajstic KM, Coates MI, Young GC, Senden TJ. 2015.** First shark from the Late Devonian (Frasnian) Gogo Formation, Western Australia sheds new light on the development of tessellated calcified cartilage. *PLOS ONE* **10(5):**e0126066 DOI 10.1371/journal.pone.0126066. |
| 16. *Squatina* sp. | **Cabrera DA, Alberto LC, Cozzuol MA. 2012.** Tridimensional Angel Shark Jaw elements (Elasmobranchii, Squatinidae) from the Miocene of Southern Argentina. *Ameghiniana* **49(1):**126_131 DOI 10.5710/AMGH.v49i1(469). |
| 17. *Squalus acanthias* | **Wilga CD, Motta PJ. 1998**. Conservation and variation in the feeding mechanism of the spiny dogfish *squalus acanthias*. The *Journal of Experimental Biology* **201(9):**1345_1358 DOI 10.1242/jeb.201.9.1345. |
| 18. *Palidiplospinax occultidens* | **Klug S, Kriwet J. 2008.** A new basal galeomorph shark (Synechodontiformes, Neoselachii) from the Early Jurassic of Europe. *Naturwissenschaften* **95(5**):443_448 DOI 10.1007/s00114-007-0341-0. |
| 19. *Synechodus dubridiensis* | **Maisey JG. 1985**. Cranial morphology of the fossil elasmobranch *Synechodus dubrisiensis*. *American Museum* novitates no. 2804. New York: American Museum of Natural History. |
| 20. *Tribodus limae* | **Lane JA, Maisey JG. 2012.** The visceral skeleton and jaw suspension in the durophagous hybodontid shark *Tribodus limae* from the Lower Cretaceous of Brazil. *Journal of Palaeontology* **86(5):**886_905 DOI 10.1666/11-139.1. |
| 21. *Onychoselache traquairi* | **Coates MI, Gess RW. 2007.** A new reconstruction of *Onychoselache traquairi,* comments on early chondrichthyan pectoral girdles and hybodontiform phylogeny. *Palaeontology* **50(6):**1421_1446 DOI 10.1111/j.1475-4983.2007.00719.x. |
| 22. *Egertonodus basanus* | **Lane JA, Maisey JG. 2012.** The visceral skeleton and jaw suspension in the durophagous hybodontid shark *Tribodus limae* from the Lower Cretaceous of Brazil. *Journal of Palaeontology* **86(5):**886_905 DOI 10.1666/11-139.1. |
| 23. *Palaeobates* *polaris* | **Romano C, Brinkmann W. 2010**. A new specimen of the hybodont shark *Palaeobates polaris* with three-dimensionally preserved Meckel's cartilage from the Smithian (Early Triassic) of Spitsbergen. *Journal of Vertebrate Paleontology* **30(6):**1673_1683  DOI 10.1080/02724634.2010.521962. |
| 24. *Tristychius* *arcatus* | **Coates MI, Tietjen KO, Aaron M, Finarelli JA. 2019**. High-performance suction feeding in an early elasmobranch. *Science Advances* **5(9**):eaax2742 DOI 10.1126/sciadv.aax2742. |
| 25. *Phoebodus* sp. | **Frey L, Coates MI, Ginter M, Hairapetian V, Rücklin M, Jerjen I, Klug C. 2019**. The early elasmobranch *Phoebodus*: phylogenetic relationships, ecomorphology, and a new time-scale for shark evolution. *Proceedings of the Royal Society* B **20191336**:1_11. |
| 26. *Orthacanthus* sp. | **Ginter M, Maisey JG. 2007**. The braincase and jaws of *Cladodus* from the Lower Carboniferous of Scotland. *Palaeontology* **50(2):**305_322 DOI 10.1111/j.1475-4983.2006.00633.x. |
| 27. *Diploselache* *woodi* | **Dick JRF. 1981**. *Diplodoselache woodi* gen. et sp. nov. an early Carboniferous shark from the Midland Valley of Scotland. *Earth and Environmental Science Transactions of The Royal Society of Edinburgh* **72(2):**99_113 DOI 10.1017/S0263593300009937. |
| 28. *Triodus* *aedorum* | **Luccisano V, Pradel A, Amiot R, Gand G, Steyer JS, Cuny G. 2021.** A new *Triodus* shark species (Xenacanthidae, Xenacanthiformes) from the lowermost Permian of France and its paleobiogeographic implications. *Journal of vertebrate Palaeontology* **41(2):**1_18 DOI 10.1080/02724634.2021.1926470. |
| 29. *Acronemus* *tuberculatus* | **Rieppel O. 1981.** The hybodontiform sharks from the Middle Triassic of Monte San Giorgio, Switzerland. *Neues Jahrbuch für Geologie und Paläontologie* **161(3):**324_353. |
| 30. *Heslerodus* *divergens* | **Hodnett JPM, Grogan E, Lund R, Lucas SG, Elliott D. 2021.** Ctenacanthiform sharks from the Late Pennsylvanian (Missourian) Tinajas member of the Atrasado formation, central New Mexico. *New Mexico Museum of Natural History and Science Bulletin* **84**:391_424. |
| 31. *Ctenacanthus* *concinnus* | **Hodnett JPM, Grogan E, Lund R, Lucas SG, Elliott D. 2021.** Ctenacanthiform sharks from the Late Pennsylvanian (Missourian) Tinajas member of the Atrasado formation, central New Mexico. *New Mexico Museum of Natural History and Science Bulletin* **84**:391_424. |
| 32. *Ctenacanthus* sp. | Unpublished material; stored at the Palaeontological Institute and Museum in Zürich, Switzerland |
| 33. *Dracopistris* *hoffmanorum* | **Hodnett JPM, Grogan E, Lund R, Lucas SG, Elliott D. 2021.** Ctenacanthiform sharks from the Late Pennsylvanian (Missourian) Tinajas member of the Atrasado formation, central New Mexico. *New Mexico Museum of Natural History and Science Bulletin* **84**:391_424. |
| 34. *Cladodoides* *wildungensis*. | **Ginter M, Maisey JG. 2007**. The braincase and jaws of *Cladodus* from the Lower Carboniferous of Scotland. Palaeontology **50(2)**:305_322 DOI 10.1111/j.1475-4983.2006.00633.x. |
| 35. *Cladodus* *elegans* | **Ginter M, Maisey JG. 2007**. The braincase and jaws of *Cladodus* from the Lower Carboniferous of Scotland. Palaeontology **50(2):**305_322 DOI 10.1111/j.1475-4983.2006.00633.x. |
| 36. *Ozarcus* *mapesae* | **Pradel, A., Maisey, J. G., Tafforeau, P., Mapes, R. H., & Mallatt, J. 2014.** A Palaeozoic shark with osteichthyan-like branchial arches. *Nature*, **509(7502):** 608-611. DOI: [10.1038/nature13195.](https://doi.org/10.1038/nature13195) |
| 37. *Akmonistion* *zangerli* | **Coates MI, Sequeira SEK. 2001.** A new stethacanthid chondrichthyan from the lower Carboniferous of Bearsden, Scotland. *Journal of Vertebrate Paleontology* **21(3):**438_459 DOI 10.1671/0272-4634(2001)021[0438:ANSCFT]2.0.CO;2. |
| 38. *Cobelodus* *aculeatus* | **Zangerl R, Case GR. 1976.** *Cobelodus aculeatus* (Cope) an anacanthous shark from Pennsylvanian Black Shales of North America. *Palaeontographica, Sonder Abdruck* **154**:107_157. |
| 39. *Ferromirum* *oukherbouchi* | **Frey L, Coates MI, Tietjen K, Rücklin M, Klug C. 2020.** A symmoriiform from the Late Devonian of Morocco demonstrates a derived jaw function in ancient chondrichthyans.  *Communications Biology* **3(1):** 1-10 DOI 10.1038/s42003-020-01394-2. |
| 40. *Chondrenchelys* *problematica* | **Finarelli JA, Coates MI. 2014.** *Chondrenchelys problematica* (Traquair, 1888) redescribed: A Lower Carboniferous, eel-like holocephalan from Scotland. *Earth and Environmental Science Transactions of The Royal Society of Edinburgh* **105(1):**35_59  DOI 10.1017/S1755691014000139. |
| 41. *Cladoselache* sp. | **Harris, J. E. 1938.** The Dorsal Spine of *Cladoselache*: The Neurocranium and Jaws of *Cladselache*. *Scientific publications of the cleveland museum of natural history* **8(1):** 1-12 |
| 42. *Maghriboselache* sp. | **Klug C, Coates M, Frey L, Greif M, Jobbins M, Pohle A, Lagnaoui A, Bel Haouz W, Ginter M. In prep.** Broad snouted cladoselachian with sensory specialisation at the base of modern chondrichthyans. |

Table 2: List of all taxa that were used in the morphometric analyses and general information about each.

| **specimen  number** | **Name** | **Locality** | **Age** | **Size** | **Class** | **Order** |
| --- | --- | --- | --- | --- | --- | --- |
| **1** | *Hangenberg black shale jaw* | Hangenberg shale morocco | latest Devonian | 1.3 cm | ?? | ?? |
| **2** | *Ischnacanthus sp.* | Midland Valley in Tealing,  Forfarshire, Scotland | Lochkovian | 4.3 cm | Acanthodii | Ischnacanthiformes |
| **3** | *Climatius reticulatus* | Tillywhandland Quarry,  Scotland | Lochkovian | ?? | Acanthodii | Climatiiformes |
| **4** | *Latviacanthus ventspilsensis* | Ventspils, Lettland | late Early Devonian | 2.0 cm | Acanthodii | Climatiiformes |
| **5** | *Ptomacanthus anglicus* | fish lens, Wayne Herbert  Quarry, Welsh Border Region | Early Devonian | 2-3 cm | Acanthodii | Climatiiformes |
| **6** | *Acanthodopsis sp.* | Northumberland Coal  Measures | Carboniferous | 3.9 cm | Acanthodii | Diplacanthiformes |
| **7** | *Tetanopsyrus lindoei* | MOTH, Canada | Lochkovian | 4.5 mm | Acanthodii | Diplacanthiformes |
| **8** | *Cheiracanthus grandispinus* | Achanarras Quarry, Caithness, Scotland | Middle Devonian | 3.7 cm | Acanthodii | Acanthodiformes |
| **9** | *Cheiracanthus murchisoni* | Tynet Burn, Scotland | Middle Devonian | 2.4 cm | Acanthodii | Acanthodiformes |
| **10** | *Halimacanthodes ahlbergi* | Gogo Formation, Canning  Basin, Western Australia. | Frasnian | 1.1 cm | Acanthodii | Acanthodiformes |
| **11** | *Acanthodes bronni* | Lebach, Saar-Nahe basin (southwesternGermany) | Early Permian,  Sakmarian–Asselian | 6.1 cm | Acanthodii | Acanthodiformes |
| **12** | *Pucapampella sp.* | Belén Formation  (La Paz region, Bolivia) | Emsian | 6.4 cm | Chondrichthyes |  |
| **13** | *Gladbachus adentatus* | Lower Plattenkalk, Unterthal, Bergisch Gladbach (Germany) | Upper Givetian,  Upper Middle Devonian | 28.9 cm | Chondrichthyes |  |
| **14** | *Kathemacanthus rosulentus* | MOTH, Canada | Lochkovian | 1.7 cm | Chondrichthyes,  (Elasmobranchs?) | incertae sedis |
| **15** | *Gogoselachus lynbeazleyae* | Gogo Formation, Canning  Basin, Western Australia. | Frasnian | 5 cm | Chondrichtyhes,  (Elasmobranchs?) | incertae sedis |
| **16** | *Squatina sp.* | Puerto Madryn Formation, northeastern Chubut Province, Argentina | Miocene | 9.7 cm | Chondrichthyes,  Elasmobranchs | Squatiniformes |
| **17** | *Squalus acanthias* | East Sound and Upright  Head off Orcas Island in the San Juan Islands, Washington, USA. | recent |  | Chondrichtyhes,  Elasmobranchs | Squatiniformes |
| **18** | *Palidiplospinax occultidens* | Lyme Regis (England) | Sinemurian, Jurassic | 4.8 cm | Chondrichthyes,  Elasmobranchs | Synechodontiformes |
| **19** | *Synechodus dubrisiensis* |  | Late Cretaceous |  | Chondrichthyes,  Elasmobranchs | Synechodontiformes |
| **20** | *Tribodus limae* | upper Romualdo member, Santana Formation, Chapada do Araripe, Ceara´ Province, northeastern Brazil | Early Cretaceous | 4.5 cm | Chondrichthyes,  Elasmobranchs | Hybodontiformes |
| **21** | *Onychoselache traquairi* | Mumbie Quarry, Glencartholm Wood, Scotland | Vise´an (Holkerian) | 1.9 cm | Chondrichthyes, Elasmobranchs | Hybodontiformes |
| **22** | *Egertonodus basanus* | ?? | ?? | 16.8 cm | Chondrichthyes, Elasmobranchs | Hybodontiformes |
| **23** | *Palaeobates polaris* | ‘fish horizon’ of Spitsbergen (Svalbard archipelago, Arctic Norway) | Smithian | 11 cm | Chondrichthyes, Elasmobranchs | Hybodontiformes |
| **24** | *Tristychius arcuatus* | Wardie Beach locality of  Edinburgh, Scotland | Middle Mississipian,  Viséan (Asbian) | 3.2 cm | Chondrichthyes, Elasmobranchs | Hybodontiformes |
| **25** | *Phoebodus sp.* | Morocco | Devonian | ?? | Chondrichtyhes,  Elasmobranchs | Phoebodontiformes |
| **26** | *Orthacanthus sp.* | ?? | Perm | ?? | Chondrichthyes, Elasmobranchs | Xenacanthiformes |
| **27** | *Diplodoselache woodi* | Oil Shale Groups, Edinburgh | Lower Carboniferous,  Visean | 15.4 cm | Chondrichthyes, Elasmobranchs | Xenacanthiformes |
| **28** | *Triodus aeduorum* | Muse OSB, Autun Basin  (Saône-et-Loire), France | lower Asselian  (lowermost Permian) | 8.3 cm | Chondrichthyes, Elasmobranchs | Xenacanthiformes |
| **29** | *Acronemus tuberculatus* | Monte San Giorgio,  Kt. Tessin, Switzerland, | Middle Triassic | 6 cm | Chondrichthyes, Elasmobranchs | Ctenacanthiformes |
| **30** | *Heslerodus divergens* | ?? | ?? | 10.4 cm | Chondrichthyes,  Elasmobranchs | Ctenacanthiformes |
| **31** | *Ctenacanthus concinnus* | Cleveland shale, Ohio, U.S.A. | Upper Devonian  (Fammenian) | 31 cm | Chondrichthyes,  Elasmobranchs | Ctenacanthiformes |
| **32** | *Ctenacanthus sp.* | Morocco | Devonian | ?? | Chondrichthyes,  Elasmobranchs | Ctenacanthiformes |
| **33** | *Dracopristis hoffmanorum* | Kinney Brick Quarry  Lagerstätte in the Manzanita Mountains east of Albuquerque New Mexico | Late Pennsylvanian  (Missiourian) | 31.1 cm | Chondrichthyes,  Elasmobranchs | Ctenacanthiformes |
| **34** | *Cladodoides wildungensis* | ?? | ?? | ?? | Chondrichthyes,  Elasmobranchs | Ctenacanthiformes |
| **35** | *Cladodus elegans* | Lugton, Ayrshire, Scotland. | Lower Carboniferous  Upper Vise´an, Brigantian Stage | 39 cm | Chondrichthyes,  Elasmobranchs | Ctenacanthiformes |
| **36** | *Ozarcus mapesae* | Arkansas,United States | LowerCarboniferous | 6.8 cm | Chondrichthyes, Holocephalans | Symmoriiformes |
| **37** | *Akmonistion zangerli* | Manse Burn Formation,  Bearsden, Scotland | Serpukhovian,  Lower Carboniferous | 11.3 cm | Chondrichthyes, Holocephalans | Symmoriiformes |
| **38** | *Cobelodus aculeatus* | central basin complex  of north america | Pennsylvanian | 13.5 cm | Chondrichthyes, Holocephalans | Symmoriiformes |
| **39** | *Ferromirum oukherbouchi* | Anti-Atlas, Morocco | Late Devonian | 3.9 cm | Chondrichthyes, Holocephalans | Symmoriiformes |
| **40** | *Chondrenchelys problematica* | Mumbie Quarry, Scotland | early Carboniferous,  Vise´an (Holkerian) | 1.7 cm | Chondrichthyes, Holocephalans | Chondrenchelyformes |
| **41** | *Cladoselache sp.* | Anti-Atlas, Morocco | Late Devonian, Famennian | ?? | Chondrichthyes, Holocephalans | Cladoselachiformes |
| **42** | *Maghriboselache sp.* | Anti-Atlas, Morocco | Late Devonian, Famennian | ?? | Chondrichthyes, Holocephalans | Cladoselachiformes |
